# Mucolytic Core–Shell Particles to Boost Levofloxacin Penetration Through the Mucus Barrier in Cystic Fibrosis

**DOI:** 10.1007/s11095-026-04103-6

**Published:** 2026-04-23

**Authors:** Valentina Ruggiero, Francesca Mariano, Domenico Larobina, Gaetano D’Avino, Marco Trofa, Consiglia Tedesco, Pasquale Del Gaudio, Paola Russo

**Affiliations:** 1 Department of Pharmacy, University of Salerno, Via Giovanni Paolo II, 132, Fisciano, 84084 Italy; 2 Institute of Polymers, Composites and Biomaterials, National Reasearch Council of Italy, P.Le E. Fermi, 1, Portici, 80055 Italy; 3 Dipartimento Di Ingegneria Chimica, Dei Materiali E Della Produzione Industriale, Università Degli Studi Di Napoli Federico II, P.Le Tecchio 80, Naples, 80125 Italy; 4 Department of Chemistry and Biology “A. Zambelli”, University of Salerno, Fisciano, SA 84084 Italy

**Keywords:** antibiotics, cystic fibrosis, dry powder inhaler, mucolytics, spray drying

## Abstract

**Purpose:**

The purpose of this study was to investigate multi-fluid spray drying as a formulation strategy to engineer inhalable microparticles containing levofloxacin and mucolytic agents, and to evaluate how formulation and process parameters influence particle properties, aerosol performance, and drug release under mucus-relevant conditions.

**Methods:**

Microparticles containing levofloxacin in combination with mucolytic agents were produced using a mini spray dryer equipped with a triple-fluid nozzle. Different compositions and particle architectures were obtained by varying formulation and excipient allocation. The resulting powders were characterized in terms of morphology, bulk, tapped and true density, aerodynamic performance, and *in vitro* drug release evaluated both in the absence and presence of a mucus layer.

**Results:**

Ambroxol showed greater suitability for spray drying than N-acetylcysteine, resulting in markedly higher process yields (up to 74%). The incorporation of L-leucine as a functional excipient reduced particle agglomeration and improved powder handling and aerosolization, with fine particle fractions exceeding 38% for leucine containing formulations. Drug release experiments demonstrated that spray drying altered release behaviour under diffusion limiting conditions imposed by a mucus layer compared to dissolution under sink conditions.

**Conclusions:**

Overall, the results indicate that multi-fluid spray drying enables effective modulation of the physicochemical and aerodynamic properties of inhalable microparticles. This study provides mechanistic insight into how formulation composition and process design influence particle behaviour in mucus-relevant environments, supporting the use of this approach as a flexible platform for the development of inhalable formulations.

## Introduction

Cystic fibrosis (CF) is a fatal genetic disease that primarily affects the respiratory and gastrointestinal systems. It is caused by mutations in the gene encoding the glycoprotein cystic fibrosis transmembrane conductance regulator (CFTR). Dysfunction of CFTR alters chloride and sodium transport across cell membranes. This reduces mucus hydration, making it thicker and more viscous. This deficit in mucociliary clearance promotes airway obstruction and the development of chronic bacterial infections [1], resulting in persistent inflammation and a progressive decline in respiratory function. In addition, dense mucus represents a physical barrier that hinders the effectiveness of drug administration.

To overcome these challenges, it is crucial to develop treatments aimed at improving the delivery of drugs directly into the lungs, whose involvement represents the main cause of morbidity in CF patients [2].

In this context, inhalation therapy emerges as one of the most promising solutions, allowing the delivery of drugs directly to the site of action, thereby reducing systemic side effects [3]. In particular, Dry Powder Inhalers (DPIs) offer numerous advantages over other inhalation delivery methods, such as greater stability of the active ingredient, ease of transport and use, absence of preservatives and reduced risk of microbiological contamination [4].

Several DPI antibiotic formulations are currently available for inhalation treatment of cystic fibrosis. One such formulation is sodium cholistimethate, used for the treatment of *Pseudomonas aeruginosa* [5] and recently developed in dry powder form demonstrating comparable efficacy and safety to inhaled tobramycin (TIS) [6]. Tobramycin is also available in a DPI formulation (TIP), administered via a T-326 inhaler. This formulation has demonstrated comparable clinical efficacy to nebulization, with a better tolerability profile and patient satisfaction [7].

Despite these advances, the number of antibiotics approved for inhalation remains limited. Moreover, their efficacy can decrease over time due to bacterial resistance and the difficulty of penetrating viscous mucus, highlighting the need for new therapeutic strategies and alternative inhaled antimicrobial agents [8].

To address the challenge caused by the particularly thick and viscous mucus of CF patients, this work proposes an innovative strategy based on a *chemical boost*. Specifically, a mucolytic agent, such as N-acetylcysteine (NAC) or Ambroxol, is used to envelop a core containing the antibiotic Levofloxacin, thus improving drug penetration and distribution. Ambroxol is a mucoactive agent widely used in the treatment of respiratory diseases for its mucokinetic and mucociliary effects, which facilitate the removal of thick mucus from the airways. In addition, it possesses anti-inflammatory and antioxidant properties and stimulates lung surfactant production, contributing to improved respiratory function [9].

Levofloxacin is a broad-spectrum antibiotic commonly used for the treatment of bacterial infections in CF patients: with formulations such as Quinsair, approved by the EMA (European Medical Agency) for the treatment of chronic respiratory infection by Pseudomonas aeruginosa in adults with CF [10].

The aim of this research is to develop and characterize inhalable dry powders obtained by spray drying using a triple-fluid nozzle, which allows the precise placement of the active ingredients in the particle core or shell to be modulated according to the desired formulation strategy. Spray drying represents an effective one-step technique to produce inhalable particles with well-defined technological properties, such as morphology, size and density, which can be modified by optimizing the process parameters [11]. To improve the aerodynamic characteristics and stability of the powders, a functional excipient, L-leucine, was also added. This amino acid improves particle aerosolization by reducing cohesive and adhesive forces between particles, as it tends to accumulate on their surface. Furthermore, it has been demonstrated that L-leucine can recrystallize during the spray drying process, forming a protective layer on the particle surface that protects it from moisture [12].

The present study focuses on physicochemical and formulation aspects, without directly addressing biological mucolytic activity, and systematically investigates the influence of feed composition and processing parameters on particle physicochemical properties and aerodynamic performance relevant to deep lung deposition.

## Materials and Methods

Levofloxacin was kindly donated by Genetic SpA (Fisciano, Italy). N-acetylcysteine (NAC) was provided from Sigma-Aldrich (Milan, Italy). Ambroxol was procured from TCI chemicals (Tokyo, Japan), l-leucine was provided from Sigma-Aldrich (Milan, Italy). HCl 1 N was supplied from VWR Chemicals (Rosny-sous-Bois, France). Clear and colorless gelatine capsules size 3 were procured from Farmalabor (Canosa di Puglia, Italy). The inhaler device, Monodose DPI RS01 model 7, was kindly offered by Plastiape SPA (Lecco, Italy). Water was purified by reverse osmosis (Milli-Q, Millipore, France). For the dissolution study, mucus was obtained from the gastric mucosa of pigs provided by a nearby slaughterhouse. In detail, porcine gastric mucus (PGM) is extracted from the stomachs of freshly slaughtered pigs and used directly, without any purification steps. The stomachs are first opened, and the inner surface is rinsed with deionized water to remove debris. Sections of roughly 10 × 10 cm^2^ are then cut out and stored frozen until required. Before each experiment, one of these sections is allowed to thaw at room temperature and the mucus is collected by gently scraping it off the surface using a spatula.

### Microparticles Preparation

Microparticles were produced through spray drying technology with a Mini Spray Dryer B-290 (Buchi Laboratoriums-Tecnik, Flawil, Switzerland) in the triple-fluid nozzle configuration. Mucolytic agents were introduced into the outer channel of the nozzle, while Levofloxacin was pumped through the inner channel. To favor efficient shell formation and particle coating, the outer feed was delivered at a higher flow rate (1.56 mL·min⁻^1^) than the inner feed (1.04 mL·min⁻^1^).

A stepwise one-factor-at-a-time approach was adopted to isolate the effect of key formulation variables and facilitate interpretation of structure–property relationships. Formulation compositions and process parameters were selected based on preliminary screening and literature data, with the aim of exploring the influence of drug ratio and excipient allocation rather than performing a full experimental design. In particular, drug concentrations were chosen to ensure complete solubilization while maintaining sufficient solid content for particle formation. The L-leucine levels (7.5–10% w/w) were selected based on its well-established role in improving powder dispersibility and aerosolization performance.

For particles containing NAC as mucolytic agent, N-acetylcysteine (3 and 5% w/v), and Levofloxacin (3 and 5% w/v), were dissolved in distilled water until complete solubilization. In some batches (Table I), to improve the solubility of Levofloxacin, few drops of 1 M hydrochloric acid were added. L-leucine was added only to the external feed as a dispersibility enhancer to improve powder flowability and reduce particle aggregation. The feed solutions were dried under the following conditions: inlet temperature 120°C, drying air flow rate 500 L/min, aspiration rate 25–35 m^3^/h, air pressure 7 atm (Table I). The outlet temperature ranged from 72°C to 77°C, depending on the composition of the feed solutions and the inlet temperature.

**Table I:** Feed Composition and Operating Parameters of Batches Containing Spray-Dried Microparticles of N-acetylcysteine and Levofloxacin

Batch	N-acetylcysteine out (% w/v)	Levofloxacin in (% w/v)	HCl	L-leucine^*^out (%w/w)	Aspiration (m^3^/h)
LENAC_a	3.0	3.0	11 GTT	-	35
LENAC_b	3.0	3.0	13 GTT	-	28
LENAC_c	3.0	3.0	-	-	28
LENAC_d	5.0	3.0	-	-	28
LENAC_e	3.0	5.0	-	-	28
LENAC_f	3.0	5.0	-	-	25
LENAC_g	3.0	5.0	-	10.0	25

^*^L-leucine to drug ratio

For Ambroxol containing microparticles, the mucolytic agent and the antibiotic drug were tested at two different concentrations (1.5 and 2.5% w/v), dissolving them in distilled water until complete solubilization. L-leucine was added as a functional excipient to both the inner (Levofloxacin) and outer (Ambroxol) feed solutions. In each feed, L-leucine was incorporated at either 7.5% or 10% w/w, calculated relative to the mass of the drug present in that specific feed. This approach ensured the presence of L-leucine in both compartments of the triple-fluid nozzle system, with a proportion that directly depended on the drug concentration of the corresponding feed (Table I). Moreover, since the outer feed was pumped at a higher flow rate (1.56 mL·min⁻^1^ *vs* 1.04 mL·min⁻^1^ inner), identical % w/w values resulted in different absolute leucine masses delivered to the spray-drying droplets.The operating parameters were as follows: inlet temperature 120°C, aspiration rate 28 m^3^/h, inlet pressure 7 bar and feed rates 1.56 mL/min for Ambroxol and 1.04 mL/min for Levofloxacin (Table II). The outlet temperature ranged from 81°C to 82°C.

**Table II:** Feed Composition for Spray-Dried Microparticles of Ambroxol and Levofloxacin

Batch	Ambroxol out (% w/v)	Levofloxacin in (% w/v)	L-leucine^*^(% w/w)	L-leucine (mg)
AMB	1.5	-	-	**-**
AL	1.5	1.5	-	-
AL_7.5l	1.5	1.5	7.5 in 7.5 out	37.0 in 56.5 out
AL_10l	1.5	1.5	10.0 in 10.0 out	49.5 in 75.0 out
A2L_7.5l	1.5	2.5	7.5 in 7.5 out	61.8 in 56.5 out
A2L_10l	1.5	2.5	10.0 in 10.0 out	82.5 in 75.0 out
2AL_7.5l	2.5	1.5	7.5 in 7.5 out	37.1 in 93.8 out

^*^L-leucine to drug ratio

All spray-dried powders were collected and stored at room temperature.

### Levofloxacin Quantification

For drug content evaluation in spray-dried powders and for *in vitro* aerodynamic studies, Levofloxacin was quantified by UV detection (Evolution 201, Thermo Fisher Scientific, Spectral, Ozzano dell’Emilia, Bologna, Italy) at a wavelength of 287 nm, using 1 cm SUPRASIL® quartz cell (Hellma 100-QS, HELLMA Italia srl, Milan, I). The analytic method was validated using standard solutions of Levofloxacin in water in the range of 1.83–18.3 μg/ml (*y* = 0.0758*x* + 0.0298; *R*^2^ = 0.9942). For release studies, Levofloxacin quantification was performed at a wavelength of 331 nm, using the same quartz cell. In this case, the analytical method was validated using standard solutions in 0.05 M phosphate buffer (pH 7.4), in the concentration range of 6.43–51.4 µg/mL (*y* = 0.0317x + 0.0006; *R*^2^ = 0.9999).

### Spray Drying Yield

After the powder was obtained using the Spray Dryer, it was collected and stored in glass vials at room temperature. Each batch was weighed to determine the percentage yield of the process using the following formula:

1 $$YIELD = \frac{mass\;of\;recovered\;powder \left(g\right)}{mass\;of\;powder\;in\;the\;feed\;liquid\;(g)} \times 100$$

### Particle Size Distribution

The particle size distribution of microparticles was determined using a light-scattering laser granulometer equipped with a tornado powder dispersing system (LS 13 320 Beckman Coulter Inc., FL, USA). The LS 13 320 uses a 5 mW laser diode with a wavelength of 750 nm and reverse Fourier optics incorporated in a fibre optic spatial filter and binocular lens systems. The particle size distributions were generated by instrument software using a *Fraunhofer Model*. The tornado module leads to a dispersion similar to the one achieved when the samples are run wet, without using any solvent which can alter powder surface properties [13]. Samples were charged into a plastic cylinder to obtain an obscuration value between 4 and 8%.

Results were expressed as *d*_50_ and span, defined as [*d* (90) − *d* (10)/*d* (50)], where *d*(10), *d*(50) and *d*(90) indicate diameters at the 10th, 50th and 90th percentiles of the particle size distribution, respectively.

### Microparticle Morphology

Morphology of microparticles was examined using a scanning electron microscope (SEM) Zeiss EVO MA10 with a secondary electron detector (Carl Zeiss SMT AG, München-Hallbergmoos, Germany), operating at 14 kV, equipped with a Leica EMSCD005 metallizator producing a deposition of a 200–440 Å thick gold layer [14].

### Bulk, Tapped, True Density and Carr's Index

Bulk density (*ρ*_*b*_) and tapped density (*ρ*_*t*_) of the spray-dried powders were measured as described elsewhere [15]. Briefly, powders were loaded into a bottom-sealed 1 ml plastic syringe (Terumo Europe, Leuven, Belgium) capped with laboratory film (Parafilm® “M”, Pechiney Plastic Packaging, Chicago, IL, USA) and tapped on a hard bench until no change in the volume of the powder was observed. Since a custom syringe-based procedure rather than the standard pharmacopeial test was performed, absolute Carr’s Index values cannot be directly compared to pharmacopeial reference limits; however, the measurements are internally consistent and allow relative comparison among the prepared batches.

In detail, the bulk and tapped densities were calculated from the net weight of the plastic syringe content divided by the powder volume in the syringe before and after tapping, respectively. Carr's Index was defined as Eq. 2:

2 $$Car{r}{\prime}s\;Index \left(\%\right)= \frac{\rho t-\rho b}{\rho t}\times100$$

Experiments were performed in triplicate.

True density of powders was measured using a helium pycnometer (Micrometrics AccuPyc II 1340 Norcross, GA, USA) with a 1 cm^3^ sample cup. Mean values and standard deviation were obtained from 10 measurements for each sample.

### Solid-State Characterization

Differential scanning calorimetry (DSC) and powder X-ray diffraction (PXRD) analysis were performed to evaluate the solid-state properties of the spray-dried formulations and assess potential changes in crystallinity induced by the process.

#### Differential Scanning Calorimetry (DSC)

DSC analysis was performed using an indium-calibrated Mettler Toledo DSC 822e (Mettler Toledo, OH, USA). Accurately weighed samples (4–5 mg) (MTS Mettler Toledo microbalance, OH, USA) were placed in a 40 μL aluminium pans, which were sealed and pierced prior to analysis, and heated up to 350°C at a heating rate of 10°C/min.

#### Powder X-Ray Diffraction (PXRD) Analysis

Approximately 200 mg of each powder sample were measured by PXRD using a Bruker D2 PHASER diffractometer equipped with LYNXEYE detector and Cu-Kα wavelength (1.5418 Å). Each pattern was collected for approximately 30 min with a 0.01° increment.

### Aerodynamic Properties

Aerodynamic properties of the powders produced were evaluated *in vitro* using the SSGI and the monodose DPI RS01 model 7. 30 ml and 7 ml water were introduced in the lower and upper stages of the SSGI, respectively. Clear and colorless gelatine capsules, size 3, were hand filled with 15–30 mg (± 0.5 mg) of microparticles, according to powder density. To ensure the powder release, each capsule was introduced into the device and pierced twice. For the deposition experiment, the vacuum pump operated at a flow rate of 60 (± 5) l/min for 5 s. The deposited powder was recovered for quantification, washing each impingement chamber of the SSGI with water. The emitted dose (ED) was gravimetrically determined and expressed as percentage of powder exiting the device *vs* amount of powder introduced into the capsule. The Fine Particle Fraction (FPF), expressed as a percentage, was defined as a ratio of the drug characterized by particles with an aerodynamic diameter smaller than 6.4 μm which passed into the lower impingement chamber *vs* total drug charged into the capsules.

### *In vitro* Drug Release and Diffusion Across a Mucus Layer

**Fig. 1:**
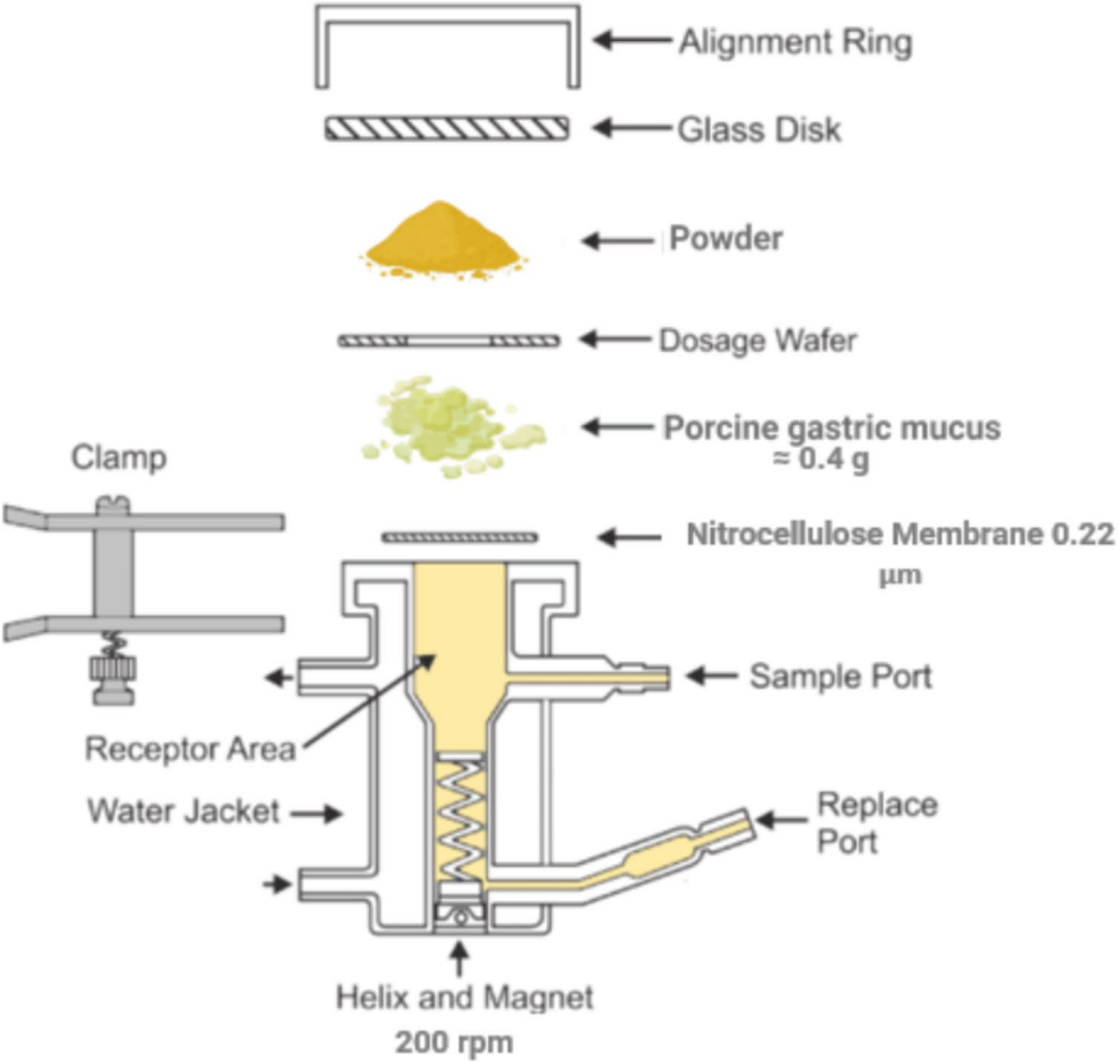
Schematic Representation of the Franz Diffusion Cell Assembled in a Vertical Configuration. A Layer of Porcine Gastric Mucus is Applied Between the Membrane and the Powder to Mimic a Mucosal Barrier.

*In vitro* drug release and diffusion through mucus were monitored by means of Franz-type vertical diffusion cells (Hanson research corporation, CA, USA). The cell system temperature was kept constant at 37°C throughout the experiment by recirculating water from a thermostatically controlled bath. Continuous stirring at 200 rpm was provided by Teflon-coated stirring bars placed in the receptor compartment. Firstly, experiments were conducted with Franz cells in a standard configuration, spreading the powder directly on the membrane. The receptor compartment was filled with 7 ml of a 0.05 M phosphate buffer (pH 7.4) and a nitrocellulose membrane (size pores: 0.22 μm), previously set with phosphate buffer, was applied between the two compartments (permeation area 1.77 cm^2^). Approximately 5 mg of pure levofloxacin were accurately weighed and placed directly onto the membrane surface within the dosage wafer. For formulation tests, an amount of formulation corresponding to 5 mg of levofloxacin was applied, calculated based on the active content in the formulation. The dosage wafer was subsequently sealed using spring clips and laboratory film (Parafilm®). In the second set of experiments, a thin layer (1,5 mm) of porcine gastric mucus was interposed between the nitrocellulose membrane and the drug formulation (Fig. 1). Samples (500 μl) were withdrawn at defined time intervals (15, 30, 60, 120 180, 240, 300, 360 and 420 min) inserting the same volume of warmed buffer. Levofloxacin was quantified by UV detection (Evolution 201, Thermo Fisher Scientific, Spectral, Ozzano dell’Emilia, Bologna, Italy) at a wavelength of 331 nm.

## Results and Discussions

### Dry Powders Production and Characterization

The present study provides a physicochemical and formulation-based investigation of spray-dried Ambroxol–Levofloxacin microparticles. The observed effects are interpreted in terms of particle structure and transport behavior rather than direct biological activity.

Process yields with N-acetylcysteine were very low (Table III). Neither the addition of hydrochloric acid drops during the feed preparation to enhance Levofloxacin solubility nor the tuning of process parameters led to substantial improvements in yield. Some of the experimental batches are reported in Table III. Due to the inability to achieve adequate yields despite numerous attempts, no further characterization studies were performed on these powders.

**Table III:** Feed Composition, Spray Yield and Operating Parameters of the Batches Containing N-acetylcysteine and Levofloxacin Spray-Dried Microparticles (mean ± SD, *n* = 3)

Batch	N-acetylcysteine out (% w/v)	Levofloxacin in (% w/v)	HCl	L-leucine^*^out (%w/w)	Aspiration (m^3^/h)	T Inlet (°C)	Yield (%)
LENAC_a	3.0	3.0	11 GTT	-	35	120	2.1 ± 1.1
LENAC_b	3.0	3.0	13 GTT	-	28	120	4.1 ± 2.3
LENAC_c	3.0	3.0	-	-	28	120	22.8 ± 1.4
LENAC_d	5.0	3.0	-	-	28	120	2.6 ± 2.4
LENAC_e	3.0	5.0	-	-	28	120	27.7 ± 1.2
LENAC_f	3.0	5.0	-	-	25	120	36.9 ± 1.1
LENAC_g	3.0	5.0	-	10.0	25	120	17.34 ± 2.3

^*^L-leucine to drug ratio

The low spray drying yield observed in NAC-containing batches could be related to the physicochemical properties of N-acetylcysteine. NAC is highly hydrophilic which may lead to increased stickiness and adherence to the drying chamber walls, thus reducing powder recovery. It is worth noting that NAC has a relatively low melting point (~ 110°C), which may further contribute to its poor processability via spray drying. Under the applied inlet temperature of 120°C, NAC may partially melt or undergo thermal softening, leading to sticky particle formation and severe wall deposition. Combined with its hydrophilic and hygroscopic nature, this thermal sensitivity likely played a key role in the lack of powder recovery observed, both when NAC was spray-dried alone and in combination with levofloxacin.

Better results in terms of process yields were obtained using Ambroxol as mucolytic agent (Table IV). A further increase in yield was observed with higher concentrations of L-leucine, with values ranging from 41.2% to 74.2%, highlighting the versatility of this excipient in improving process performance across different formulations. L-leucine is known to form a hydrophobic surface layer on particles during the spray drying process: this layer reduces particle adhesion to the drying chamber walls, thereby minimizing losses and enhancing the recovery of the final product.

**Table IV:** Feed Composition, Spray Yield and Size Distribution of the Batches Containing Ambroxol and Levofloxacin Spray-Dried Microparticles (mean ± SD, *n* = 3)

Batch	Ambroxol out (%w/v)	Levofloxacin in (%w/v)	L-Leucine^*^(%w/w)	Yield (%)	D_50_ (μm) and (SPAN)
AMB	1.5	-	-	41.2 ± 1.1	3.41 ± 0.11
AL	1.5	1.5	-	-	-
AL_7.5l	1.5	1.5	7.5 in 7.5 out	53.1 ± 2.1	2.63 ± 0.12
AL_10l	1.5	1.5	10.0 in 10.0 out	68.5 ± 0.7	3.11 ± 0.03
A2L_7.5l	1.5	2.5	7.5 in 7.5 out	59.3 ± 2.3	2.73 ± 0.12
A2L_10l	1.5	2.5	10.0 in 10.0 out	66.5 ± 4.1	3.15 ± 0.02
2AL_7.5l	2.5	1.5	7.5 in 7.5 out	61.6 ± 1.7	2.82 ± 0.12
2AL_10l	2.5	1.5	10.0 in 10.0 out	74.2 ± 1.8	2.84 ± 0.03

^*^L-leucine to drug ratio

Particle size analysis revealed that spray drying successfully produced micronized powders with a d_50_ ranging from 2.63 μm to 3.41 μm, which was consistent across all batches. No significant effect of L-leucine content on particle diameter was observed, and the particle size remained well-suited for inhalation administration (Table IV).

The incorporation of L-leucine in Ambroxol-Levofloxacin feeds markedly influenced the physicochemical and aerodynamic properties of the resulting microparticles.

A clear interpretation of the density data emerges when considering both the total mass of L-leucine incorporated in each batch and, critically, its distribution between the inner (core-forming) and outer (shell-forming) feeds. Bulk density shows a consistent reduction when higher amounts of leucine were used, especially when the excipient is predominantly supplied through the outer feed (e.g., 2AL_10l). This pattern aligns with leucine-driven surface corrugation, which disrupts particle packing and lowers pour density. Correspondingly, these batches also exhibit the largest increases in tapped density, indicating that the highly corrugated particles undergo substantial rearrangement and compaction upon tapping. Thus, bulk and tapped density trends are primarily governed by surface-related effects, and correlate best with the outer-feed leucine content.

In contrast, true density reveals a different dependency (Table V). The lowest value is observed for A2L_10l, the batch with the highest inner-feed leucine mass. Since leucine introduced internally is more likely to contribute to internal porosity during droplet drying and solute redistribution, this results in a less dense matrix. Batches where leucine is mainly supplied through the outer feed show pronounced surface roughness and reduced bulk density but comparatively moderate reductions in true density, consistent with a predominantly surface effect.

**Table V:** Bulk, Tapped and True Density and Carr’s Index of the Spray-Dried Microparticles Containing Ambroxol and Levofloxacin

Batch	Ambroxol out (% w/v)	Levofloxacin in (% w/v)	L-leucine^*^(% w/w)	L-leucine (mg)	ρᵦ (mg/ml)	ρₜ (mg/ml)	Carr’s Index (%)	True Density (g/cm^3^)
AL_7.5l	1.5	1.5	7.5 in 7.5 out	37.0 in 56.5 out	290.6 ± 4.2	453.1 ± 8.0	41.0 ± 8.5	1.71 ± 0.02
AL_10l	1.5	1.5	10.0 in 10.0 out	49.5 in 75.0 out	241.5 ± 1.0	483.0 ± 1.9	50.0 ± 0.0	1.69 ± 0.01
A2L_7.5l	1.5	2.5	7.5 in 7.5 out	61.8 in 56.5 out	307.9 ± 2.1	552.9 ± 51.3	39.5 ± 0.7	1.75 ± 0.01
A2L_10l	1.5	2.5	10.0 in 10.0 out	82.5 in 75.0 out	254.7 ± 15.9	499.2 ± 17.5	49.0 ± 1.4	1.53 ± 0.00
2AL_7.5l	2.5	1.5	7.5 in 7.5 out	37.1 in 93.8 out	306.0 ± 0.6	600.2 ± 15.5	49.0 ± 1.4	1.61 ± 0.02
2AL_10l	2.5	1.5	10.0 in 10.0 out	49.5 in 112.5 out	264.9 ± 0.9	589.3 ± 15.7	55.0 ± 2.1	1.59 ± 0.04

^*^L-leucine to drug ratio

In conclusion, bulk and tapped density behaviour reflects the surface architecture driven by outer-feed leucine, whereas true density captures modifications of the internal structure governed by inner-feed leucine. These complementary trends highlight the importance of reporting absolute leucine content per feed and clarifying its distribution in multi-fluid spray drying processes, as they directly influence powder cohesiveness, porosity, and ultimately aerosol performance.

**Fig. 2:**
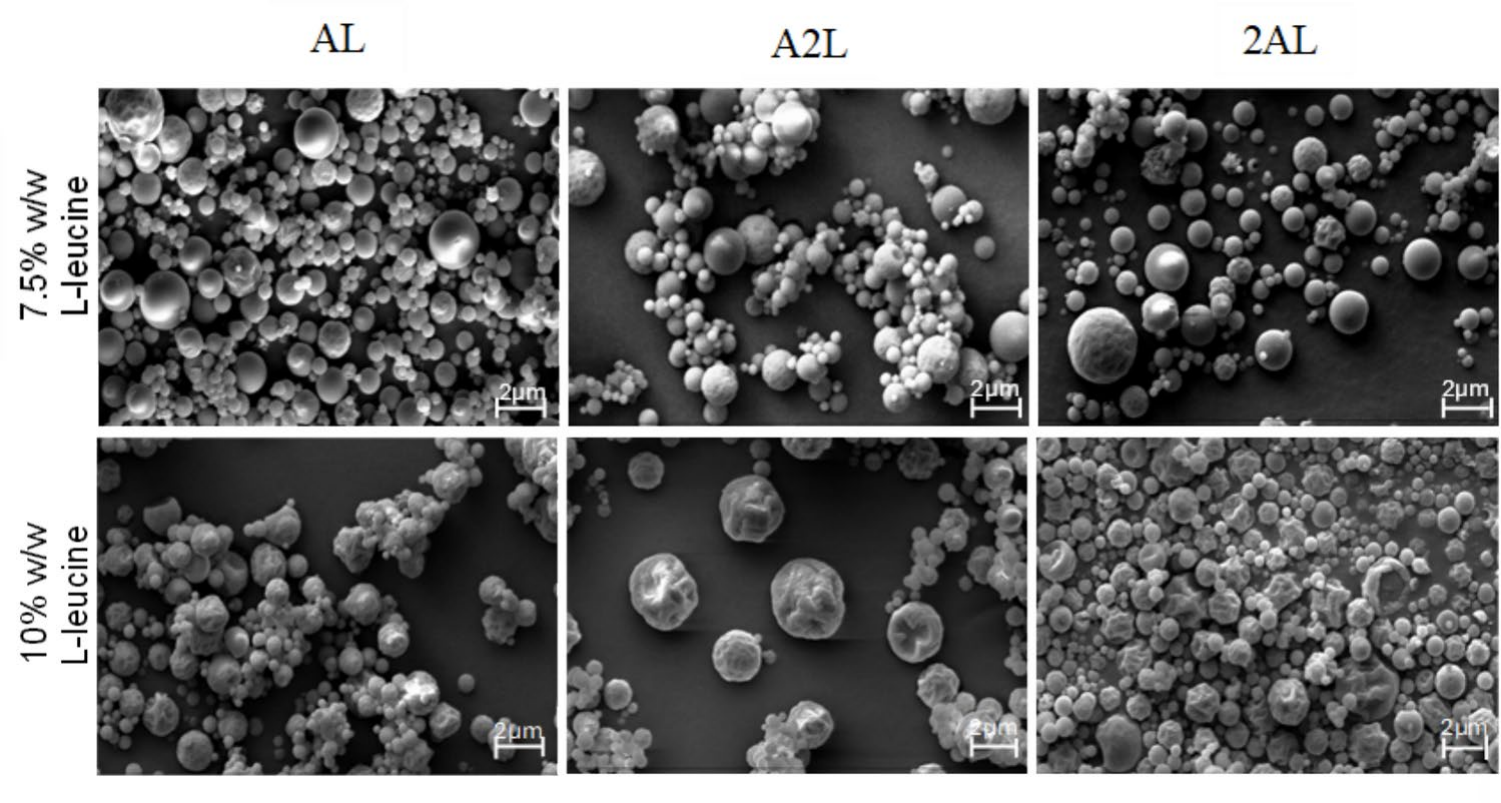
SEM Images of AL, A2L, and 2AL with 7.5% and 10% L-leucine.

Following morphological analysis with Scanning Electron Microscopy (SEM) (Fig. 2), the particles produced with 7.5% and 10.0% w/w of L-leucine were found to be well-separated, demonstrating L-leucine’s effective role in reducing particle aggregation. However, at a higher concentration, the particles exhibit a visibly more corrugated surface, with areas where L-leucine appears to have precipitated on the surface.

This morphology can be explained by considering both the Péclet number (Pe) and the different solubility of the components. A high Péclet number, linked to the rapid evaporation of the solvent, favors the migration of less soluble solutes, such as L-leucine, towards the surface of the particles during drying. L-leucine, with a solubility of approximately 22 mg/mL in water at 25°C [16], and known for its high surface activity and rapid crystallization, tends to precipitate early at the particle surface. In contrast, levofloxacin, being slightly more soluble, may remain predominantly distributed within the particle core.

Solid-state characterization by XRPD and DSC was performed to investigate the physical state of active ingredients after spray drying.

**Fig. 3:**
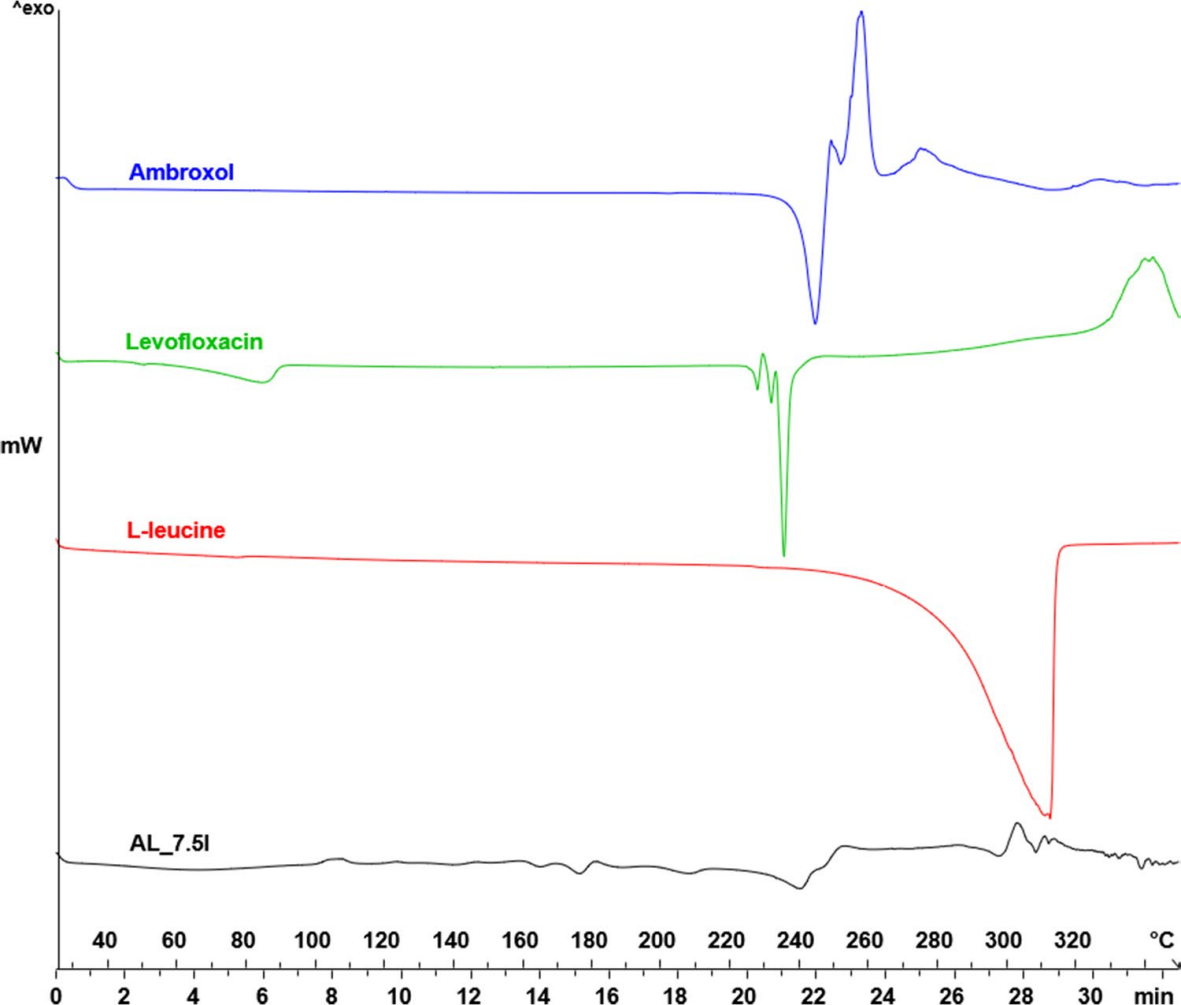
Differential Scanning Calorimetry Thermograms of Ambroxol (blue), Levofloxacin (green), L-leucine (red), and AL_7.5 l (black).

The DSC thermograms of raw materials and of a spray-dried formulation (AL_7.5 l) are reported in Fig. 3.

The raw components exhibit distinct thermal transitions characteristic of their crystalline nature: in particular, Ambroxol shows a pronounced endothermic peak at approximately 250°C, corresponding to its melting point. In agreement with literature data, this melting event was followed by an exothermic peak attributed to thermal decomposition of the compound [17]. Levofloxacin displays a sharp endothermic event at approximately 230–235°C, indicative of its crystalline form [18]. Finally, the excipient L-leucine presented a melting event at approximately 315°C [19]. The DSC thermogram of a spray-dried formulation reported as example (AL_7.5 l) shows a markedly different thermal behavior. The characteristic melting peaks of Ambroxol, Levofloxacin, and L-leucine are no longer clearly detectable, and only broad and low-intensity thermal events are observed in the 230–300°C temperature range. The disappearance of the distinct melting transitions suggests a significant reduction in crystallinity and indicates that the components are predominantly present in a disordered or amorphous state following spray drying.

**Fig. 4:**
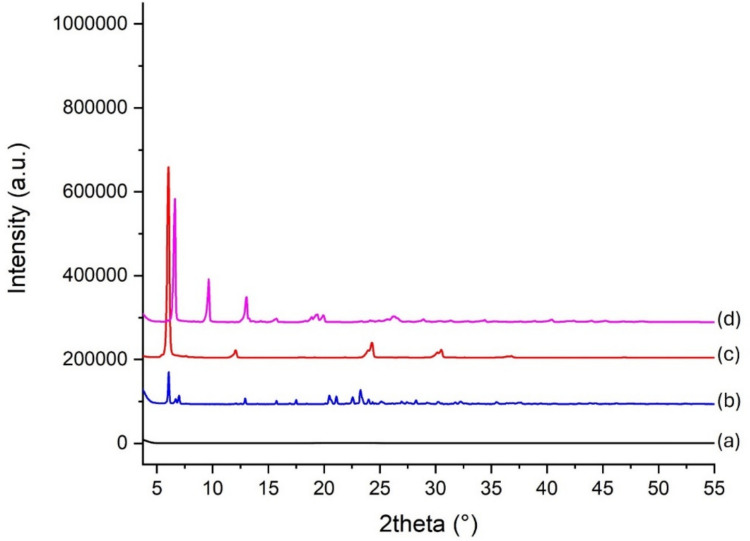
Comparison Between PXRD Patterns of (**a**) Sprayed Batch Sample (black); (**b**) Ambroxol (blue); (**c**) L-leucine (red); (**d**) Levofloxacin (magenta).

These findings are consistent with the PXRD patterns, where the raw materials exhibit sharp diffraction peaks indicative of their high crystallinity, while the spray-dried formulation shows the absence of distinct peaks (Fig. 4), confirming that the spray-drying process induces amorphization.

The combined DSC and XRPD results therefore confirm that the spray drying process inhibits crystal growth and promotes the formation of an amorphous solid dispersion of the components.

Regarding the aerodynamic characterization, the Single Stage Glass Impinger (SSGI) was used to evaluate the behavior of the best powder during inhalation. The Emitted Dose (ED) was found to be greater than 98% in all batches, an excellent result that confirms the effective dosing of the powder from the capsules, regardless of the composition. The use of higher L-leucine concentrations (10.0% w/w) had a positive impact on particle distribution, increasing the Fine Particle Fraction (FPF) up to 38% for the powder containing the same concentration of drug into the liquid feeds (Table VI).

**Table VI:** Composition and Aerodynamic Properties of Powders

Batch	Ambroxol out (% w/v)	Levofloxacin in (% w/v)	L-Leucine^*^(% w/w)	L-leucine (mg)	Fine Particle Fraction (%)	Emitted Dose (%)
AL_7.5l	1.5	1.5	7.5 in 7.5 out	37.0 in 56.5 out	37.3 ± 2.1	99.9 ± 0.1
AL_10l	1.5	1.5	10.0 in 10.0 out	49.5 in 75.0 out	38.5 ± 0.5	99.3 ± 0.2
A2L_7.5l	1.5	2.5	7.5 in 7.5 out	61.8 in 56.5 out	21.2 ± 2.2	99.3 ± 0.4
A2L_10l	1.5	2.5	10.0 in 10.0 out	82.5 in 75.0 out	34.1 ± 1.3	99.5 ± 0.2
2AL_7.5l	2.5	1.5	7.5 in 7.5 out	37.1 in 93.8 out	25.3 ± 1.7	98.8 ± 0.5
2AL_10l	2.5	1.5	10.0 in 10.0 out	49.5 in 112.5 out	35.4 ± 3.2	± 0.3

^*^L-leucine to drug ratio

### Drug Release and Diffusion Through Mucus

For the drug to be effective, once deposited in the respiratory tract, it must be released from the formulation and dissolve in the pulmonary fluids.

This process may be compromised in pathological conditions such as cystic fibrosis, which is characterized by the presence of particularly dense and viscous mucus that limits drug access to the respiratory epithelium.

This can alter the timing and efficiency of the release of the active ingredient from the inhaled formulation. In this study, drug release and diffusion through mucus was evaluated using a method based on a Franz-type diffusion apparatus in the vertical configuration, applying the powder directly onto a synthetic membrane or the porcine gastric mucus layer.

The Franz cell model employed here allows evaluation of drug release under diffusion-limiting conditions but does not enable direct assessment of mucolytic activity or mucus structural modification.

Although this model does not allow the biological effect of Ambroxol to be assessed, it does provide useful information on the ability of particles to cross a viscous barrier and the dynamics of release under standardized conditions.

**Fig. 5:**
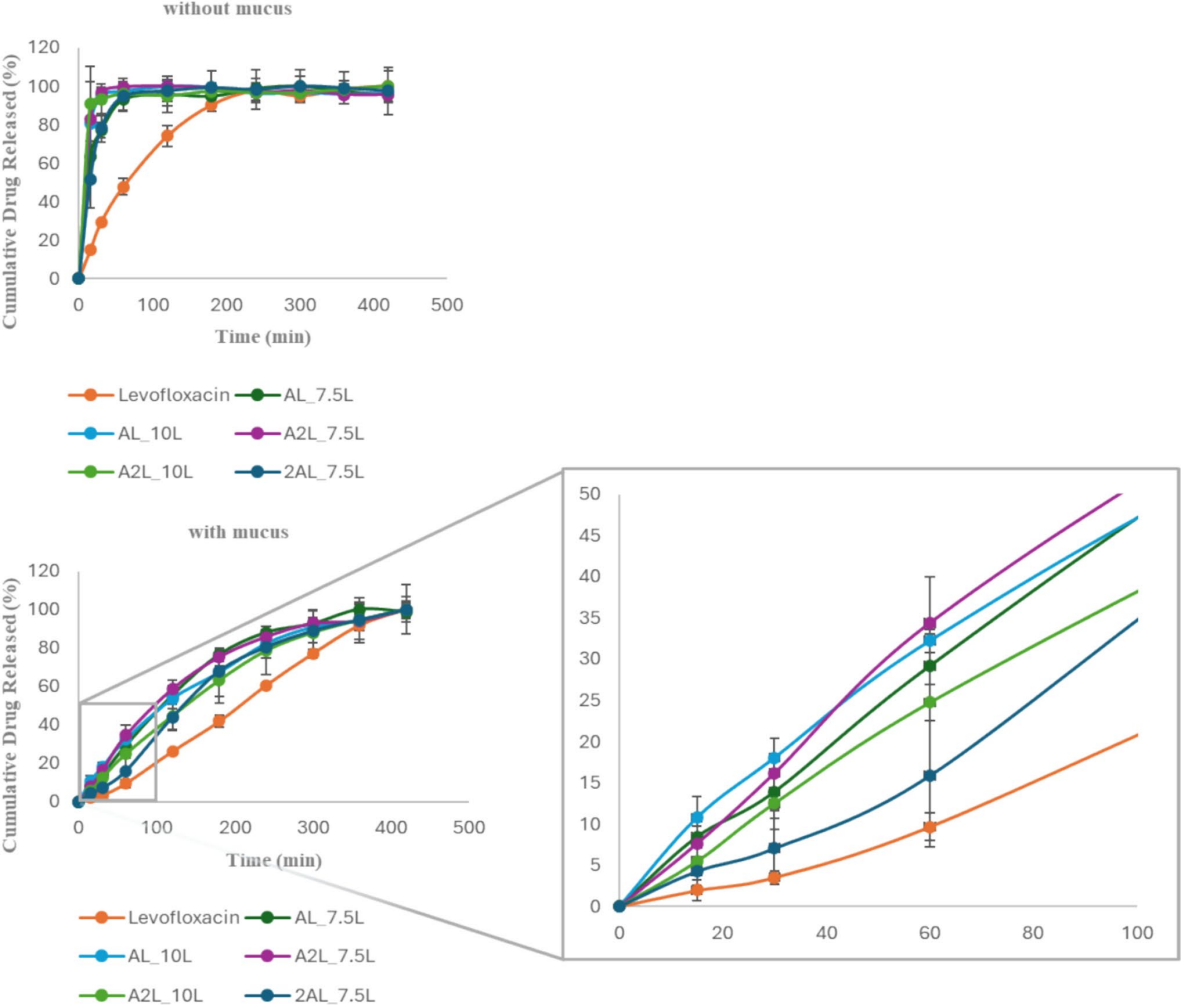
Dissolution Profiles of Formulations in Buffer (**a**) and Across a Porcine Mucus Layer (**b**), with Enlarged View.

Specifically, as a first experimental step, the dissolution capacity of unprocessed Levofloxacin was evaluated in the absence and in the presence of mucus (Fig. 5, a).

The profile obtained without mucus shows that the maximum release of Levofloxacin from a non respirable powder is reached after approximately 4 h. From the graph, it is evident how the mucus presence influences the quantity of active ingredient detected into the receiving compartment. The mucus layer slowed the dissolution and diffusion of levofloxacin, acting as a physical barrier.

After preliminary tests conducted on unprocessed Levofloxacin, both in the absence and presence of mucus, the study focused on evaluating batches obtained according to the core–shell engineering.

In the absence of mucus, all formulations obtained by spray drying showed relatively rapid release of levofloxacin, reaching maximum values within the first 60 min, unlike unprocessed and non respirable levofloxacin, which has a slower release profile. This behavior can be attributed to several factors induced by the spray drying process, including the reduction in particle size and the consequent increase in specific surface area, which promotes greater contact between the active ingredient and the dissolution medium. In addition, the process may cause partial amorphization of Levofloxacin, resulting in an increase in solubility compared to the crystalline form of the raw material [20].

However, all formulations exhibited a slower release of levofloxacin than in tests conducted in the absence of a mucosal barrier. In particular, formulations with high outer-feed leucine show a slower initial diffusion through mucus, suggesting that extensive surface deposition of leucine may transiently impede wetting and mass transfer; however these same formulations preserve excellent aerosol performance. Thus, while outer-feed leucine clearly improves aerosolization by reducing inter-particle cohesion, its net effect on mucus dissolution and diffusion may be biphasic: initial delay caused by a surface barrier followed by rapid release once the shell is disrupted or hydrated.

Overall, these results indicate that the presence of a mucus layer fundamentally alters the dissolution behaviour, highlighting the importance of evaluating inhalable formulations under diffusion-limiting conditions that better reflect the pulmonary environment.

Although direct surface characterization was not performed, the influence of formulation design on particle morphology and performance suggests the presence of compositional gradients induced by the multi-fluid spray drying process.

## Conclusions

This study investigated the use of triple-fluid spray drying to produce microparticles for pulmonary delivery, combining levofloxacin with mucolytic agents within engineered particulate systems. The formulation and process design enabled modulation of particle morphology, powder properties, and aerodynamic performance, providing insight into how particle architecture influences behaviour in mucus-relevant environments.

The use of ambroxol resulted in higher process yields compared to N-acetylcysteine, indicating greater suitability for spray-drying under the investigated conditions. The incorporation of L-leucine contributed to the formation of well-dispersed microparticles with reduced agglomeration and improved aerosolization, as reflected by fine particle fractions exceeding 38%.

Release studies performed in the absence and presence of a mucus layer showed that spray drying altered the release behaviour of levofloxacin relative to the raw material, highlighting the impact of particle engineering on drug transport across diffusion-limiting barriers. These effects can be attributed to changes in particle morphology, surface characteristics, and solid-state properties induced by the spray drying process.

All together, these results demonstrate that triple-fluid spray drying represents a flexible platform for tailoring the physicochemical and aerodynamic properties of inhalable microparticles. However, it must be highlighted that these findingsb, ased on non-viable porcine mucus, does not allow for an evaluation of the biological effect of ambroxol, which acts primarily in the presence of living tissue. To clarify the role of ambroxol in mucosal barrier penetration, further investigation in more physiologically relevant models is required.

## Data Availability

The datasets generated and analysed during the current study are available within the article. Additional data are available from the corresponding author on reasonable request.
